# Diffuse Large B-Cell Lymphoma in Human T-Lymphotropic Virus Type 1 Carriers

**DOI:** 10.1155/2012/262363

**Published:** 2011-11-09

**Authors:** Brady E. Beltran, Pilar Quiñones, Domingo Morales, Jose C. Revilla, Jose C. Alva, Jorge J. Castillo

**Affiliations:** ^1^Department of Oncology and Radiotherapy, Edgardo Rebagliati Martins Hospital, Lima, Peru; ^2^Department of Pathology, Edgardo Rebaglati Martins Hospital, Lima, Peru; ^3^Department of Oncology, Daniel Alcides Carrion Hospital, Lima, Peru; ^4^Division of Hematology and Oncology, The Miriam Hospital, Brown University Warren Alpert Medical School, Fain Building, 164 Summit Avenue, Providence, RI 02906, USA

## Abstract

We describe the clinical and pathological characteristics of seven patients who were human T-lymphotropic virus type 1 (HTLV-1) carriers and had a pathological diagnosis of *de novo* diffuse large B-cell lymphoma. Interestingly, three of our cases showed positive expression of Epstein-Barr-virus, (EBV-) encoded RNA within the tumor cells indicating a possible interaction between these two viruses. Furthermore, our three EBV-positive cases presented with similar clinical characteristics such as early clinical stage and low-risk indices. To the best of our knowledge, this is the first case series describing the characteristics of HTLV-1-positive DLBCL patients. The potential relationship between HTLV-1 and EBV should be further explored.

## 1. Introduction

Diffuse large B-cell lymphoma (DLBCL) is the most common variant of non-Hodgkin lymphoma (NHL) accounting for approximately 30% of the NHL cases worldwide [1]. Previous reports have associated certain viral infections with the development of DLBCL. For example, HIV-infected individuals have a higher risk of developing DLBCL than the general population. Additionally, the most recent WHO classification has included a provisional entity, EBV-positive DLBCL of the elderly, which seems to be associated with an aggressive clinical course and worse outcome [2]. In general, it is thought that HIV-infected and other immunocompromised individuals are more likely to develop EBV-positive DLBCL.

The human T-lymphotropic virus type 1 (HTLV-1) is a retrovirus regarded as the pathogenic agent for adult T-cell lymphoma/leukemia (ATLL) [3]. HTLV-1 is endemic in Japan, the Melanesian Islands, the Caribbean, South America, the Middle East, and parts of Africa. The prevalence of HTLV-1 in Europe and USA is <1%. However, in Peru, up to 3% of the healthy adult population carries HTLV-1 [4, 5]. Chronic HTLV-1 infection has been associated with immunosuppression and an increased risk of developing other benign and malignant conditions [6]. 

As the association between HTLV-1 infection and DLBCL has not been previously evaluated, in this study, we aimed to describe the clinical and pathological characteristics of HTLV-1-positive patients with a pathological diagnosis of DLBCL.

## 2. Materials and Methods

### 2.1. Case Selection

Cases with a pathological diagnosis of *de novo* DLBCL and concurrent positive serology for HTLV-1 were identified from the medical oncology consultation files at the Edgardo Rebagliati Martins and Jose Alcides Carrion Hospitals, both located in Lima, Peru, from January 2000 to December 2010. Patients with a diagnosis of HIV infection, transformed, primary cutaneous, or primary central nervous system (CNS) DLBCL were excluded. All cases had HTLV-1 detected in serum by ELISA and/or Western blot techniques. Clinical and laboratory information was obtained through medical chart review, after approval of this study by the Institutional Review Board at each center.

### 2.2. Pathological Evaluation

Routine hematoxylin and eosin-stained sections were prepared from formalin-fixed, paraffin-embedded tissue blocks. Immunohistochemical analysis included a broad panel of antibodies against CD45 (Dako, Carpinteria, Calif; dilution 1 : 400), CD20 (clone L26, Dako; dilution 1 : 100), MUM1/IRF4 (clone MUM1p, Santa Cruz Biotechnology, Santa Cruz, Calif; dilution 1 : 200), bcl-6 (Dako; dilution 1 : 10), and CD10 (Novocastra; dilution 1 : 10). CD10, bcl-6, and MUM1/IRF4 were considered positive if expressed by >30% of the tumor cells. For the detection of EBV, we used a chromogenic *in situ* hybridization (CISH) technique to evaluate the presence of EBV-encoded RNA (EBER; Dako). Cases showing EBER nuclear expression in >10% of the tumor cells were considered positive. The presence of proviral HTLV-1 DNA was evaluated in the tissue blocks using a polymerase chain reaction (PCR) technique as previously described [7].

## 3. Results

Seven consecutive patients with a pathological diagnosis of *de novo* DLBCL and positive serology for HTLV-1 were identified. One case has been previously reported [8]. Complete clinical and pathological data are shown in Tables 1 and 2, respectively. Clinically, the male-to-female ratio was 3 : 4, with a median age of 63 years (range 45–85 years). Three patients (43%) had early stage, and 4 patients (57%) presented with advanced stages. Low or low-intermediate IPI scores were seen in 3 patients (43%) and high or high-intermediate in 4 cases (57%). Three patients (43%) were treated with R-CHOP and 4 (57%) with CHOP alone. Six patients (86%) obtained a CR after chemotherapy. After 24 months of followup, 2 patients (29%) have died, and the median overall survival (OS) has not been reached. The estimated 2-year OS is 71%. Pathologically, all the cases (100%) had strong expression of CD20 and diffuse large cell morphology. CD10 was positive in 1 out of 5 patients tested (20%), bcl-6 was negative in all cases tested (0/5; 0%), and MUM1/IRF4 was positive in 4 out of 5 cases tested (80%). Three cases (42%) were positive for EBER by CISH. PCR used to detect proviral HTLV-1 DNA in the tumor samples was negative.

## 4. Discussion

In this paper, we present a case series of 7 HTLV-1 carriers who have developed *de novo* DLBCL. A most salient point is that three of our cases (42%) demonstrated the presence of EBV genome in the tumor cells. Although this could suggest a high incidence of EBV positivity in HTLV-1 patients with DLBCL, if we consider that the incidence of EBV-positive DLBCL has been reported in the range of 3–15% [9–12], given the small number of cases, it remains speculative. Another important aspect of our study is that these patients were negative to other viruses such as HIV and hepatitis B and C, which could also induce immunosuppression, and have been associated with the development of specific types of lymphoma. Few cases of lymphomas arising in HTLV-1 carriers have been previously published [13–16].

EBV is a recognized oncovirus with B-cell lymphotropism. EBV attaches to CD21 preparing the B-lymphocyte for EBV infection. EBV infection will promote an increased production of IL-6 and EBV-associated mRNAs promoting a blastic transformation. EBV is then inserted into the nucleus of the B cell where it acquires a circle-shaped configuration. EBV nuclear antigens are the first to be produced after infection, which are essential for immortalization of the cell and upregulation of the expression of other molecules and genes such as latent membrane proteins (LMPs) and C-MYC. LMPs increase expression of bcl-2 and drive the cell into a latent state, which is maintained by the production of EBV-encoded RNA. Hence, EBV-infected B cells enter an apparent resting phase; however, due to their activated phenotype, they are more prone to develop oncogenic changes [17]. 

HTLV-1, on the other hand, is a retrovirus that infects a wide variety of cells (lymphocytes, monocytes, and fibroblasts) [18]. An important HTLV-1-associated viral protein denominated Tax is a necessary first step in oncogenesis. Tax increases proliferation of virus-infected cells by accelerating all the phases of the cell cycle and renders the affected cells susceptible to a series of genetic and epigenetic changes [19]. The expression of Tax, however, wears out as cells acquire the ability to proliferate independently. Due to its prolonged latency period of decades, HTLV-1-infected cells are more susceptible to acquire malignant phenotypes in a multistep process. Previous studies have indicated a strong association between HTLV-1 infection and the development of ATLL. Interestingly, the frequency of primary malignant neoplasms in HTLV-1 carriers is higher than in HTLV-1-seronegative cases [6], suggesting the oncogenic power of HTLV-1 goes beyond lymphoma and leukemogenesis. Although HTLV-1 has not been associated with the development of B-cell lymphomas, HTLV-1 carriers with B-cell lymphoma tend to have worse prognosis [20]. 

However, as we suggest in the present paper, there could be a potential lymphomagenetic interaction between EBV and HTLV-1. In a previous paper in patients with ATLL, Ueda et al. indicated that coinfection with HTLV-1 and EBV may induce a more extensive organ involvement through the enhanced expression of adhesion molecules via IL-4 signaling [21]. Similarly, Ogata et al. found a subclinical reactivation of EBV in ATLL patients undergoing chemotherapy [22]. Furthermore, several papers on EBV-associated lymphoproliferative disorders (LPDs) seen in ATLL patients have been reported in the literature. Amano et al. described a case of EBV-associated primary CNS lymphoma arising in a patient with ATLL and was explained by a suppression of the immune system by HTLV-1 [23]. Tanaka et al. described a case of acute type ATLL complicated by the development of EBV-associated LPD which was likely responsible for the patient's demise [24].

Theoretically, HTLV-1 infection can cause immunosuppression *via* T-cell dysfunction and promote reactivation of EBV, which in turn will induce B-cell proliferation and lymphomagenesis. The identification of 3 HTLV-1 carriers with EBV-positive DLBCL in our study may suggest the immunosuppression induced by HTLV-1 could be implicated in the pathophysiology of this rare lymphoma. Additionally, our 3 EBV-positive DLBCL cases had similar clinical characteristics (i.e., early disease, low IPI scores, and achievement of CR with chemotherapy). Hence, we postulate an interesting hypothesis about a potential pathogenetic relationship between HTLV-1 and EBV. 

We understand the limitations of small retrospective case series such as ours, in terms of selection bias. However, these cases were identified from nonselected, consecutive patients with a diagnosis of DLBCL who were treated according to their diagnosis and stage with standard therapies. In order to investigate the potential relationship between HTLV-1 and EBV, large prospective cohort or population-based retrospective studies are needed.

## 5. Conclusion

In this paper, we present 7 cases of DLBCL in HTLV-1 carriers from which 3 were EBV-positive DLBCL. Likely, the interaction between EBV and HTLV-1 could promote T-cell and B-cell dysfunction as well as antiapoptosis and cell proliferation, favoring lymphomagenesis. Further studies are needed to investigate this potential relationship.

## Figures and Tables

**Table 1:** Clinical characteristics of 7 HTLV-1-positive patients with DLBCL.

Case	Sex	Age	WBC (×10^9^/L)	ALC (×10^9^/L)	Hb (g/dl)	Platelets (×10^9^/L)	Stage	IPI score	Treatment	Response	Overall survival	Outcome
(1)	M	85	5.4	1.6	13.5	145	I	1	CHOP	CR	5 months	Dead
(2)	F	54	6.1	1.5	11.9	230	II	0	CHOP	CR	44 months	Alive
(3)	M	73	5.7	2.5	12.1	293	II	1	R-CHOP	CR	19 months	Alive
(4)	M	47	4.9	1.1	11	240	IV	3	CHOP	PD	8 months	Dead
(5)	F	45	6.2	0.9	10.5	200	IV	3	R-CHOP	CR	24 months	Alive
(6)	F	65	5.4	1.3	11.1	180	III	3	CHOP	CR	48 months	Alive
(7)	F	63	7.2	1.3	10	320	IV	4	R-CHOP	CR	21 months	Alive

ALC: absolute lymphocyte count; Hb: hemoglobin; IPI: international prognostic index; M: male; F: female; CHOP: cyclophosphamide, doxorubicin, vincristine, and prednisone; R-CHOP: rituximab + CHOP; CR: complete response; PD: progressive disease; WBC: white blood cell count.

**Table 2:** Pathologic characteristics of 7 HTLV-1-positive patients with DLBCL.

Case	CD20	MUM1	CD10	BCL6	EBER	CD30
(1)	+	+	−	−	+	−
(2)	+	−	+	−	+	ND
(3)	+	+	−	−	+	ND
(4)	+	ND	ND	ND	−	−
(5)	+	+	−	−	−	ND
(6)	+	ND	ND	ND	−	ND
(7)	+	+	−	−	−	ND

EBER: EBV-encoded RNA by chromogenic* in situ* hybridization; ND: not done.
